# Supplementary material for: Dissociable Neural Representations of Adversarially Perturbed Images in Convolutional Neural Networks and the Human Brain
Source: Front Neuroinform. 2021 Aug 5;15:677925. doi: 10.3389/fninf.2021.677925 (PMC8375771; doi:10.3389/fninf.2021.677925)
Supplement: Supplementary file 1 [file Data_Sheet_1.pdf]

Supplementary Material

1 Supplementary Note

1.1 Hierarchical correspondence between AlexNet and human visual cortex only holds for AI images

Previous studies have revealed that the tuning complexity along the human ventral stream corresponds to the feature complexity from low- to high-level layers in CNNs (Guclu and van Gerven, 2015;Zhang et al., 2018). We thus investigated the hierarchical correspondence between AlexNet and the human brain under different image conditions. In each brain ROI, we calculated the proportion of vertices whose responses can be best explained by the units in each layer in AlexNet. This yielded a 1 x 8 vector of the proportion values for that brain ROI. This calculation was performed independently in each subject and in each of the two presentation trials. We could thus obtain six independent measurements (3 subjects x 2 trials) for the proportion vector in this ROI. We then averaged the proportion values of the low-level layers (i.e., layers 1&2) across subjects and trials, and also high-level layers (i.e., layers 7&8). A paired t-test was performed to examine the difference between the proportion values of the low-level and the high-level layers.

Since all brain ROIs here are early visual areas, we expected that the forward encoding models using low-level features should better characterize empirically measured cortical responses. Indeed, this pattern was observed in the AI images (Figure S1)–that is, the low-level CNN layers, compared with the high-level CNN layers, better predict the vertex responses (layers 1-2 vs. 7-8, paired t-test, $p < 0.0001$) across almost all ROIs. On the AN images, however, no significant difference was detected between the predictive power of the low-level and the high-level layers (layers 1-2 vs. 7-8, paired t-test, $p = 0.2351$). Moreover, we found that, from V1 to LO, the higher-level CNN layers predicted more and more vertices' responses (V1 vs. LO, paired t-test, $p = 0.0004$) towards the AI images. But we did not observe such trend in the AN images (V1 vs. LO, paired t-test, $p = 0.5631$). In other words, the hierarchical correspondence between AlexNet and the human brain observed in regular images also holds in AI images but not in AN images, further suggesting the idiosyncratic processing of AN images in AlexNet.

2 Supplementary Figures and Tables

2.1 Figure S1. Percentage of vertices explained by encoding models in each ROI

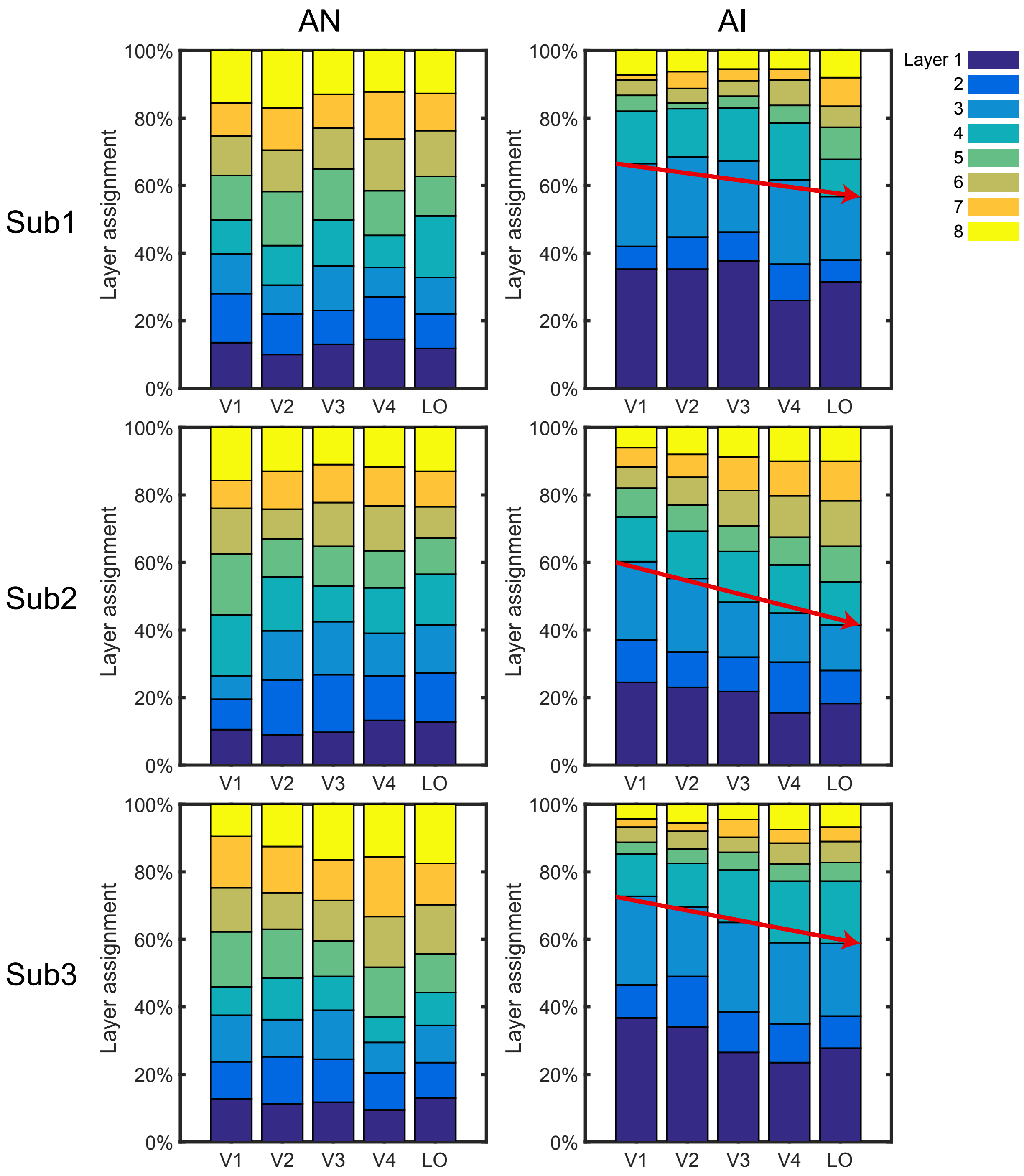

Figure S1. The percentage of vertices that can be best explained by features in each CNN layer. For the AI images, we replicate the hierarchical correspondence between AlexNet and the human brain—early visual areas can be better explained by feature activity in low-level CNN layers. This pattern is, however, not obvious for the AN images. Moreover, the proportion of vertices assigned to high-level CNN layers decreases along processing hierarchy (i.e., V1 to LO) for the AI images (indicated by the red arrows), but not for the AN images. These results indicate that the hierarchical correspondence between the AlexNet and the human brain only holds for the AI images.

2.2 Figure S2. RE-AN and RE-AI similarities with 400 vertices in each ROI

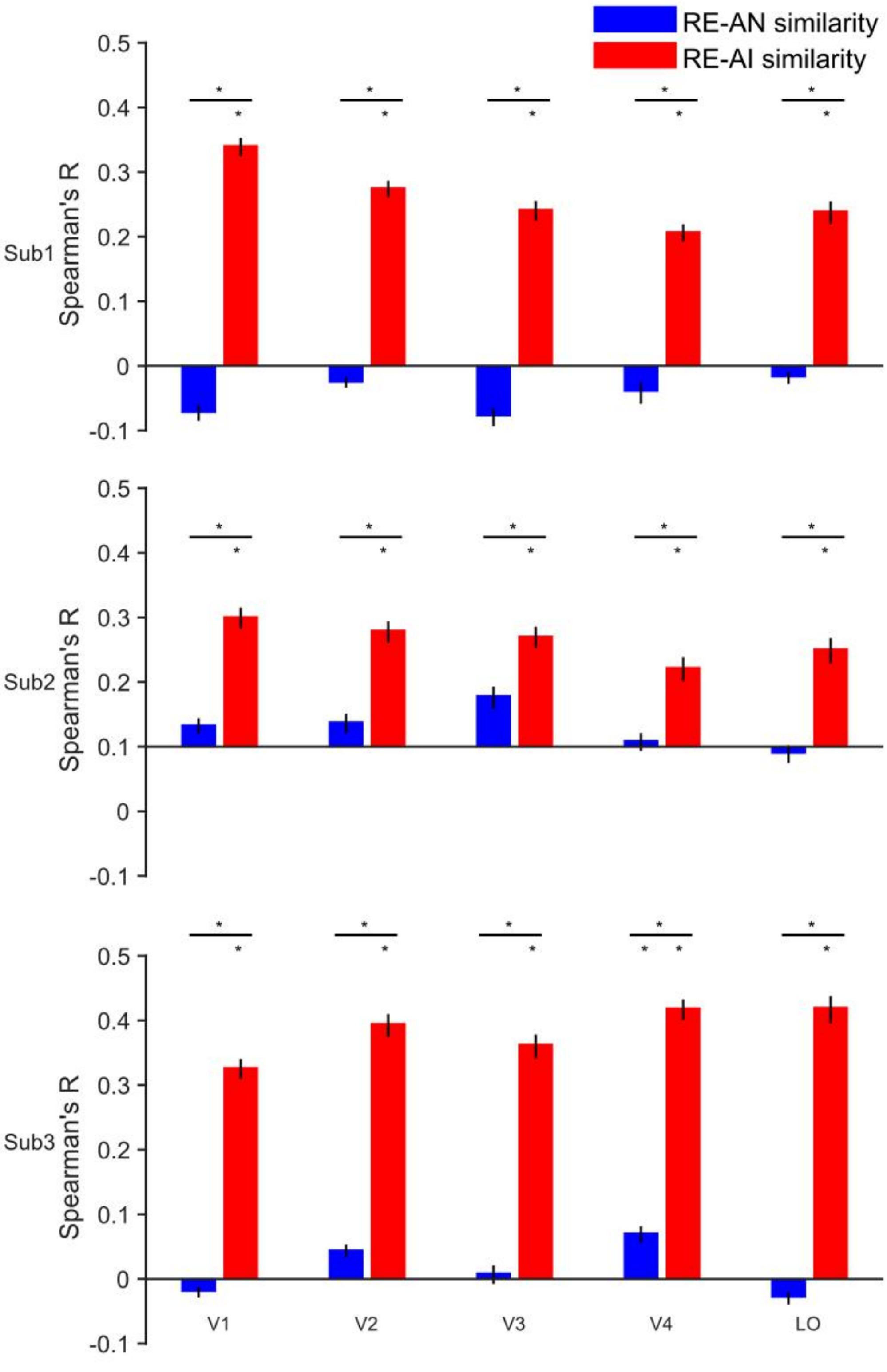

Figure S2. RE-AI and RE-AN similarity in the human brain. This figure is similar to Figure 3 except that 400 vertices are selected in each brain region.

2.3 Figure S3. Cortical topology of RE-AI and RE-AN similarities for broader regions

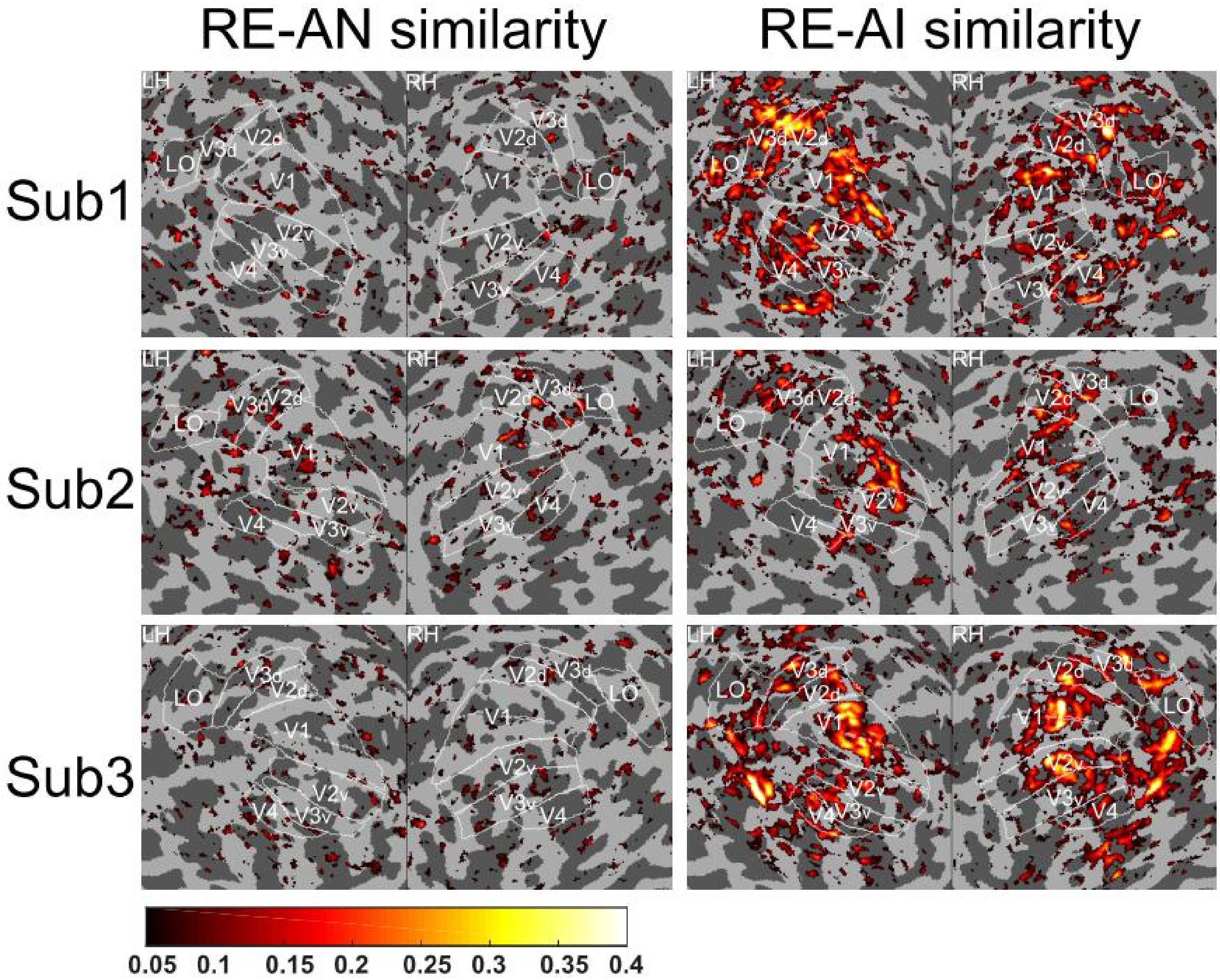

Figure S3. Cortical topology of RE-AI and RE-AN similarities for broader regions. This figure is similar to Figure 4 except that the searchlight analysis is expanded for broader regions.

2.4 Figure S4. Representational similarity analysis of AI images with over 0.99 wrong classification probability

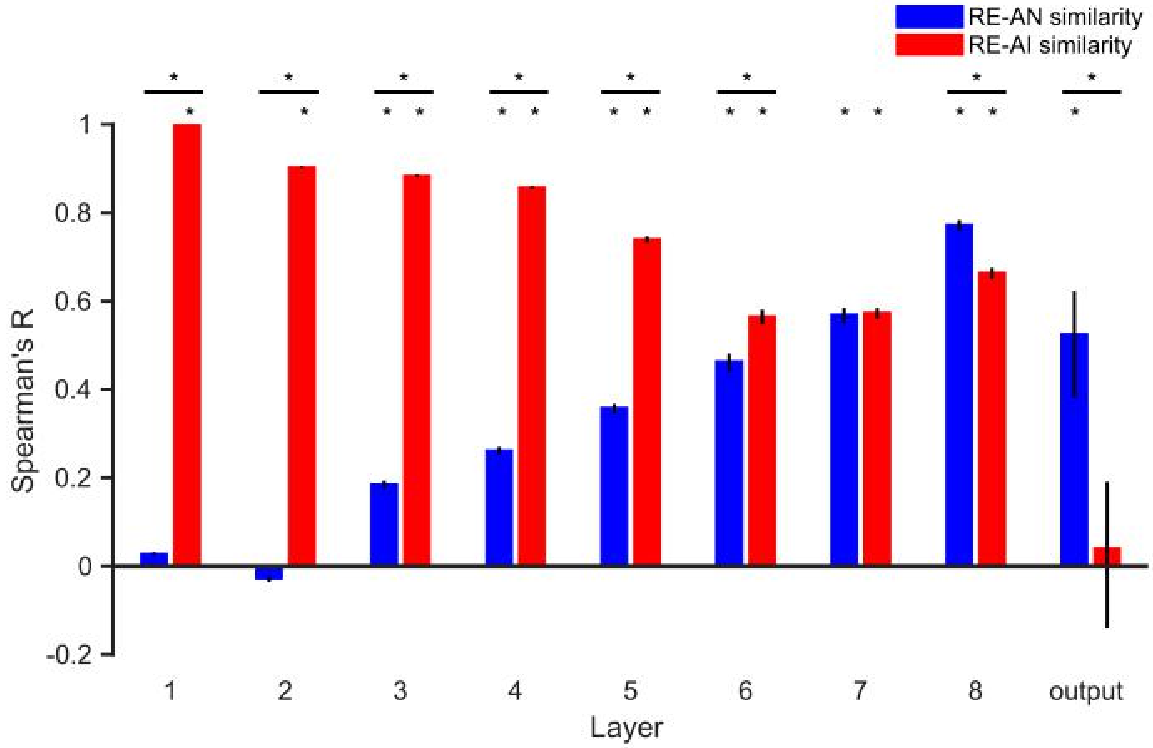

Figure S4. Representational similarity analysis of AI images with over 0.99 wrong classification probability. The analyses here are identical to the Figure 7 in the main text, except that we used the AI images that were trained to reach 0.99 probability with which they were classified to wrong categories. The overall pattern is consistent with the Figure 5. In most DNN layers, the RE-AI similarity values are almost no less than the RE-AN similarity values, indicating that the AI images cannot fool the DNN in most layers. However, the RE-AI similarity drastically drops in the output layer, indicating the potential caveat in its decision mechanisms.

2.5 Figure S5. The null hypothesis distributions for the RE-AN and the RE-AI similarity

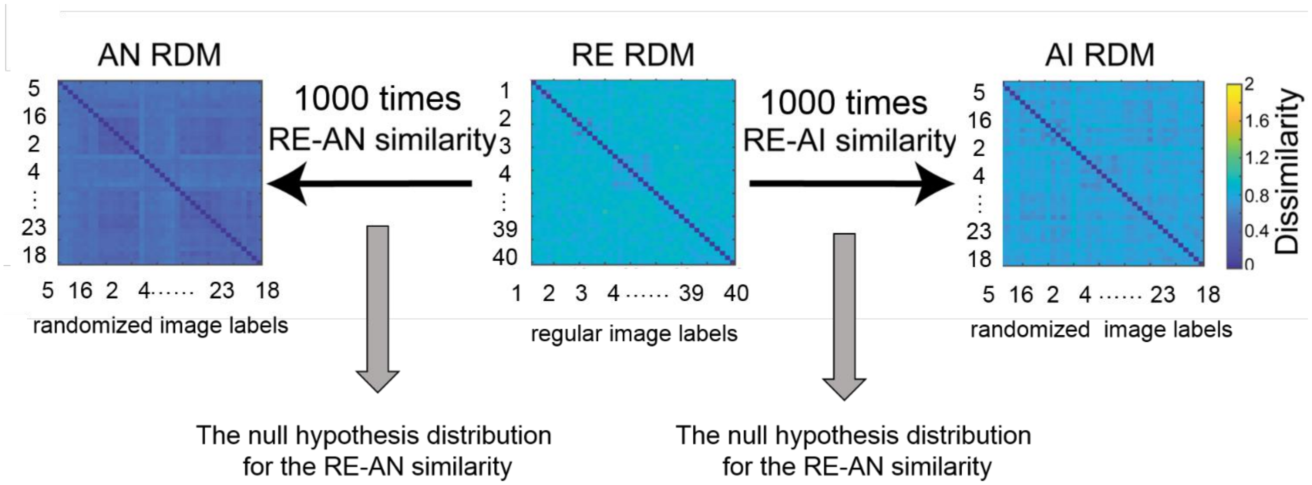

Figure S5. The null hypothesis distributions for the RE-AN and the RE-AI similarity.

2.6 Figure S6. The resampling procedures for Forward encoding modeling

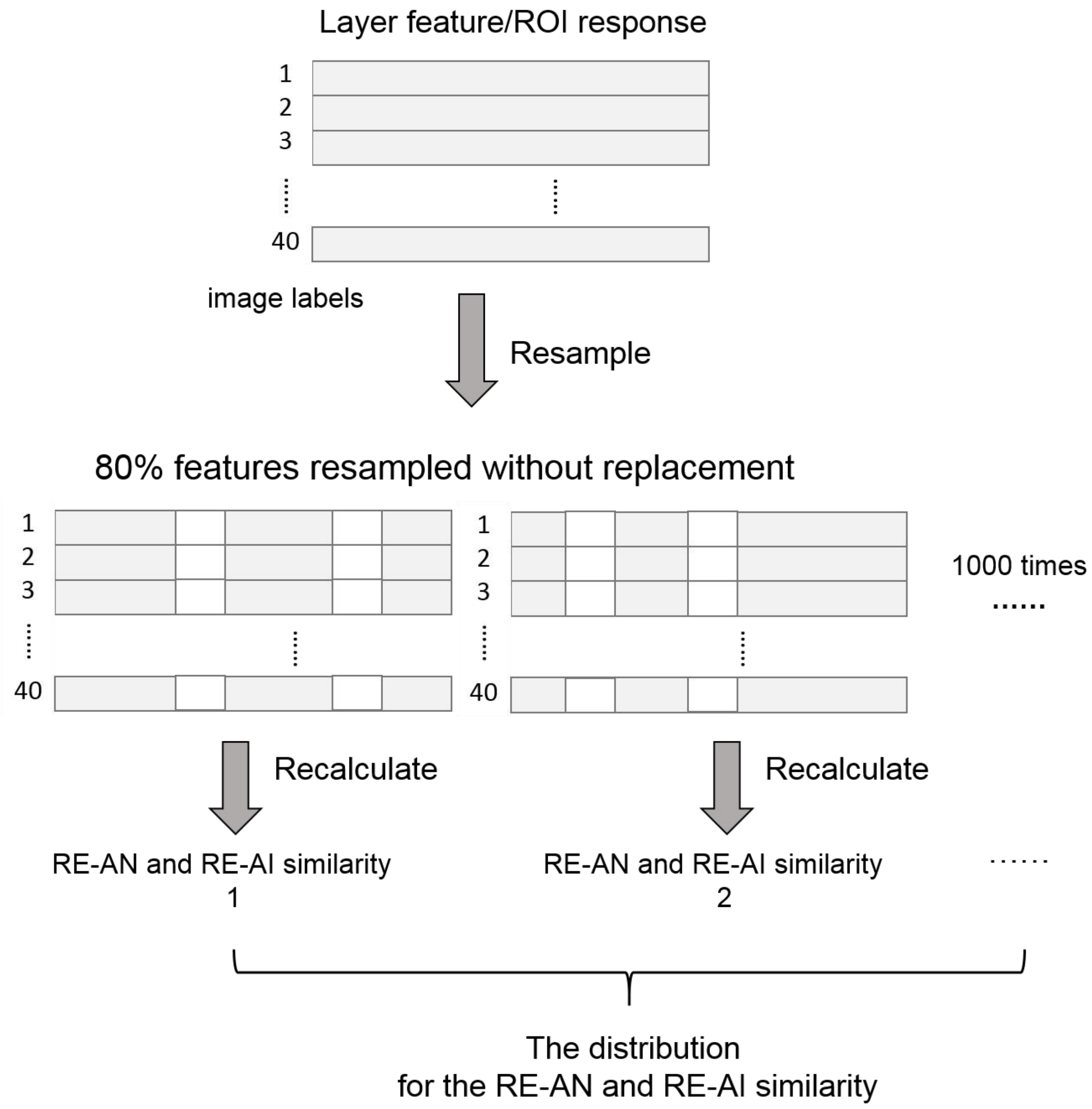

Figure S6. The resampling procedures for Forward encoding modeling.

2.7 Figure S7. The null hypothesis distributions for the RE-AN and the RE-AI similarity

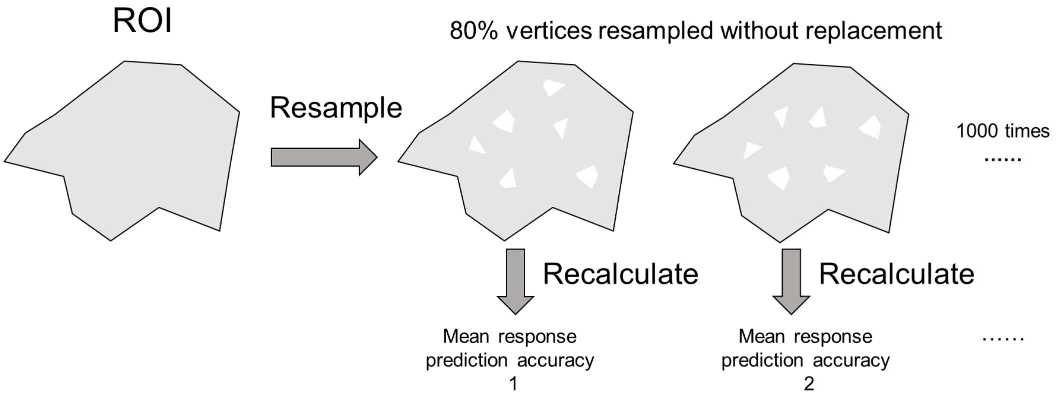

Figure S7. The null hypothesis distributions for the RE-AN and the RE-AI similarity

2.8 Table S1. Category information for the RE and corresponding AI images

Table S1. Category information for the RE and corresponding AI images.

	**RE Image**			**Corresponding AI Image**	
**Image Index**	**Class Name**	**Category Name**	**Category index in ImageNet**	**Category Name**	**Category index in ImageNet**
1		Maltese dog Maltese terrier Maltese	154	guinea pig	339
2		Blenheim spaniel	157	tennis ball	853
3		borzoi Russian wolfhound	170	llama	356
4		Ibizan hound Ibizan Podenco	174	zebra	340
5	dogs	Norwegian elkhound	175	timber wolf	270
6		Weimaraner	179	shower cap	794
7		Irish setter	214	wig	904
8		pug pug-dog	255	bath towel	435
9		goldfinch Carduelis carduelis	12	king penguin	146
10		house finch linnet Carpodacus mexicanus	13	marmoset	378
11		junco snowbird	14	ptarmigan	82
12		indigo bunting indigo finch indigo bird Passerina cyanea	15	peacock	85
13	birds	robin American robin Turdus migratorius	16	worm fence	913
14		bulbul	17	worm fence	913
15		macaw	89	agama	89
16		sulphur-crested cockatoo Kakatoe galerita Cacatua galerita	90	bee	310
17		ambulance	408	lifeboat	626
18		cab hack taxi taxicab	469	grille	582
19		fire engine fire truck	556	traffic light	921
20	cars	jeep landrover	610	projectile	745
21		Model T	662	stretcher	831
22		pickup pickup truck	718	toaster	860
23		police van	735	pool table	737

24		school bus	780	yurt	916
25	fruits	Granny Smith	949	tennis ball	853
26		strawberry	950	ice cream	929
27		orange	951	soup bowl	810
28		lemon	952	soup bowl	810
29		fig	953	plate	924
30		pineapple ananas	954	cardoon	947
31		banana	955	ear spike capitulum	999
32		pomegranate	958	ant	311
33	aquatic animals	goldfish Carassius auratus	2	banded gecko	39
34		great white shark	3	oxygen mask	692
35		electric ray	6	shovel	793
36		jellyfish	108	stove	828
37		rock beauty	393	pinwheel	724
38		anemone fish	394	jigsaw puzzle	612
39		garfish	396	American chameleon	41
40		lionfish	397	screw	784

REFERENCES

Guclu, U., and Van Gerven, M.A. (2015). Deep Neural Networks Reveal a Gradient in the Complexity of Neural Representations across the Ventral Stream. *J Neurosci* 35**,** 10005-10014.

Zhang, C., Qiao, K., Wang, L., Tong, L., Zeng, Y., and Yan, B. (2018). Constraint-Free Natural Image Reconstruction From fMRI Signals Based on Convolutional Neural Network. *Frontiers in Human Neuroscience* 12**,** 242.
